# Effect of Cu_2_O Substrate on Photoinduced Hydrophilicity of TiO_2_ and ZnO Nanocoatings

**DOI:** 10.3390/nano11061526

**Published:** 2021-06-09

**Authors:** Maria V. Maevskaya, Aida V. Rudakova, Alexei V. Emeline, Detlef W. Bahnemann

**Affiliations:** 1 Laboratory “Photoactive Nanocomposite Materials”, Saint-Petersburg State University, Ulianovskaia str. 1, Peterhof, 198504 Saint-Petersburg, Russia; maevskaya.mv@gmail.com (M.V.M.); Aida.Rudakova@spbu.ru (A.V.R.); alexei.emeline@spbu.ru (A.V.E.); 2 Institut fuer Technische Chemie, Gottfried Wilhelm Leibniz Universitaet Hannover, Callinstrasse 3, D-30167 Hannover, Germany

**Keywords:** photoinduced hydrophilicity, surface energy, heterostructures, charge transfer, work function, adsorbed water

## Abstract

The effect of a Cu_2_O substrate on the photoinduced alteration of the hydrophilicity of TiO_2_ and ZnO surfaces was studied. It was demonstrated that the formation of heterostructures Cu_2_O/TiO_2_ and Cu_2_O/ZnO strongly changed the direction of the photoinduced alteration of surface hydrophilicity: while both TiO_2_ and ZnO demonstrate surface transition to superhydrophilic state under UV irradiation and no significant alteration of the surface hydrophilicity under visible light irradiation, the formation of Cu_2_O/TiO_2_ and Cu_2_O/ZnO heterostructures resulted in photoinduced decay of the surface hydrophilicity caused by both UV and visible light irradiation. All observed photoinduced changes of the surface hydrophilicity were compared and analyzed in terms of photoinduced alteration of the surface free energy and its polar and dispersive components. Alteration of the photoinduced hydrophilic behavior of TiO_2_ and ZnO surfaces caused by formation of the corresponding heterostructures with Cu_2_O are explained within the mechanism of electron transfer and increasing of the electron concentration on the TiO_2_ and ZnO surfaces.

## 1. Introduction

Surface wettability is an important property of modern functional materials [1,2]. The self-cleaning, anti-fogging and anti-corrosive action of photoactive coatings and photocatalytic materials is based on a light-controlled surface hydrophilicity effect [3]. The photoinduced alteration of the surface wettability possesses a number of advantages compared to other methods based on the application of electric potential, mechanical stress, thermal or chemical action on the surface, etc. Photostimulated alteration of the surface hydrophilicity is easily controllable, environmentally friendly, energetically beneficial, safe and non-destructive.

Since the discovery of the effect of photoinduced superhydrophilicity for the titanium dioxide surface [4], fundamental studies of the mechanism and key factors of the process of the photoinduced alteration of the surface hydrophilicity are still in progress. Nowadays, it is established that electronic photoexcitation of the coating material plays the key role in this surface process, which is confirmed by numerous experimental data obtained by different research groups [4,5,6,7,8,9,10,11]. Particularly, it is assumed that the trapping of photo-generated charge carriers by active surface sites leads to further rearrangement of the hydroxyl-hydrated layer. As a result of such reconstruction, the surface free energy (SFE) changes. Then, the hydrophilicity of the surface changes accordingly: the higher the SFE is, the more hydrophilic the surface becomes.

Recently, we proposed a mechanism of photoinduced hydrophilic conversion, which includes elementary steps associated with the photoactivation and photodeactivation of the active surface sites responsible for the transition of the surface from one hydrophilic state to another [5,6]. It is represented as follows:

S + h(e) → S*                   *k*_1_, (1)

S* + e(h) → S                   *k*_2_, (2)

S* → H                        *k*_3_, (3)

H + e(h) → S                   *k*_4_, (4)

H → S                        *k*_5_, (5)

and a solution of the corresponding set of differential kinetic Equations (1)–(5) gives the expression for the rate of the photoinduced hydrophilic conversion (Equation (6)):

(6) ∆H(t)=H(t)−H0=(ABS0−H0)(1−e−(CB)t),

where A = *k*_1_·*k*_3_·*n*_1_, B = *k*_2_·*n*_2_ + *k*_3_ and C = *k*_1_·*k*_3_·*n*_1_ + *k*_2_·*k*_4_·*n*_2_^2^ − *k*_3_·*k*_4_·*n*_2_; *k*_i_ is the rate constant of the i-th stage; S_0_ is the initial concentration of the surface sites (S) acting as either hole or electron trap; and *n*_1_ and *n*_2_ are the surface concentrations of the photocarriers (electrons and holes), participating in activation (1) and deactivation (2) of the active surface sites.

The direction of the photoinduced alteration of the surface hydrophilicity is determined by the sign of the ΔH(t) value: for a positive value, the surface becomes more hydrophilic, and, for a negative value, it becomes more hydrophobic. Analyzing the kinetic Equation (6), one can see that the rate and direction of the process on the surface of the same material is dictated by the ratio of the concentration of photocarriers of opposite sign (*n*_1_*/n*_2_) [5,6]. Upon photoexcitation of a solid, the ratio between concentrations of electrons and holes can be changed by two means.

The first method is achieved by varying the spectral composition of the acting light, namely by the photoexcitation of the material in the region of its intrinsic or extrinsic absorption. The change in surface wettability with a change in the spectral composition of the irradiating light has been demonstrated for various coatings [5,7,9,12,13,14,15].

The second way to change the *n*_1_*/n*_2_ ratio is to create a layered heterostructured coating, the components of which form a type II heterostructure. In the literature on self-cleaning materials, the formation of composite, or heterostructured, coatings is primarily mentioned as an effective method for improving their self-cleaning properties due to the enhanced photocatalytic oxidative ability [3,16,17,18]. It is well known that the formation of the type II heterojunctions promotes the charge separation, which reduces electron–hole recombination, thus improving the photocatalytic efficiency of such systems compared to single photocatalysts [3,15,19,20,21]. At the same time, it has been shown that this approach is also promising and productive both for fundamental studies of photoinduced superhydrophilicity of the surface and for application of self-cleaning coatings with controlled wettability [5,6,22,23,24].

In this study, the effect of the Cu_2_O substrate on the photoinduced hydrophilic behavior of TiO_2_ and ZnO thin films was studied and interpreted using the proposed approach (Equations (1)–(6)). For this purpose, the “layer-by-layer” thin films of TiO_2_/Cu_2_O and ZnO/Cu_2_O, with TiO_2_ and ZnO layers on the top of the sandwich-like heterostructures, were synthesized. These pairs of photoactive metal oxides were chosen due to suitable relative alignment of their conduction and valence band edges to form type II, staggered heterojunctions: the conduction-band edge and the valence-band edge of cuprous oxide are higher in energy than the corresponding band edges of either titanium dioxide or zinc oxide. As a result, for both TiO_2_/Cu_2_O and ZnO/Cu_2_O heterostructures, electrons are confined in the titanium and zinc oxides, while holes can be accumulated in Cu_2_O. Due to such an effective charge separation, the considered composite materials have already demonstrated their high efficiency in both photoelectrochemical and photocatalytic processes [25,26,27,28,29,30,31,32,33,34,35,36,37].

In this study, we demonstrated that the formation of TiO_2_/Cu_2_O and ZnO/Cu_2_O heterojunctions drastically changes the surface hydrophilic properties of TiO_2_ and ZnO surfaces under UV irradiation. In addition, it was shown that selective photoexcitation of Cu_2_O with visible light also affects the surface hydrophilicity of the TiO_2_ and ZnO components due to electron transfer in heterostructures.

## 2. Materials and Methods

The individual ZnO and Cu_2_O films were formed by a sol-gel dip coating method (KSV Nima dip coater, Espoo, Finland) on SiO_2_-coated glass substrates to prevent the diffusion of sodium ions from the glass during thermal treatments. The velocity of withdrawing from solution was 100 mm/min for all coatings.

For the copper oxide sol, 15 mL of diethanolamine (99%, Fluka, Seelze, Germany), used as a stabilizer, were intensively stirred in 150 mL of isopropanol (99.8%, Ecos-1, Moscow, Russia), and then 14.9 g of copper (II) acetate hydrate Cu(OAc)_2_·H_2_O (99.0%, Vekton, Saint-Petersburg, Russia) were added at room temperature. A dark blue solution with a concentration of 0.5 M was kept for 24 h before further procedure [38]. To form Cu_2_O films, the obtained Cu(II)-containing layers were annealed at 300 °C on a hot plate in a nitrogen atmosphere for 40 min.

For the zinc oxide sol, 2.5 mL of ethylene glycol (99.5%, LenReactiv, Saint-Petersburg, Russia) and 10 g of zinc acetate dihydrate Zn(OAc)_2_·2H_2_O (99.0%, Vekton, Saint-Petersburg, Russia) were mixed in a round-bottomed flask and heated at 100 °C for 15 min to obtain a uniform transparent mixture. After cooling down to room temperature, 150 mL of isopropanol (99.8%, Ecos-1, Moscow, Russia) and 6.4 mL of triethylamine (99.5%, PanReac AppliChem, Darmstadt, Germany) were added to the mixture to promote the hydrolysis of the zinc acetate. Then, 0.5 mL of glycerin (99.5%, LenReactiv, Saint-Petersburg, Russia) were added dropwise to improve the film quality. The obtained clear and homogeneous solution previously stirred at 60 °C for 1 h was aged for 24 h at room temperature [39]. The formed ZnO layers were annealed at 280 °C in ambient atmosphere for 60 min.

The TiO_2_ thin film was formed by the atomic layer deposition (ALD) method (“Nanosurf” installation produced by “Nanoengineering Ltd.”, Saint-Petersburg, Russia, RC “Centre for Innovative Technologies of Composite Nanomaterials”, Research Park, Saint-Petersburg State University) on SiO_2_-coated glass substrate. Titanium tetrachloride (CAS №7550-45-0, quality level MQ200, Merck, Darmstadt, Germany) and deionized water were used as titanium precursor and hydrolysis agent, respectively. The deposition was carried out on the substrate at 200 °C.

The “layer-by-layer” TiO_2_/Cu_2_O and ZnO/Cu_2_O systems were formed by deposition of TiO_2_ and ZnO layers, respectively, on a thin Cu_2_O film formed as described above.

The surface morphology and film thickness of all synthesized coatings were explored by scanning electron microscopy (Zeiss Supra 40 VP system, Oberkochen, Germany). The smoothness of the film surface was assessed by the AFM method. X-ray diffraction measurements with Bruker “D8 DISCOVER” high-resolution diffractometer (CuKa X-ray radiation, within the angle range of 20° ≤ 2θ ≤ 80° with a scanning speed 5.0°/min, Germany) were used for the crystal phase determination. Structural reference data were taken from the ICSD database. The transmittance spectra were recorded in the 250–800 nm spectral range at ambient conditions using Lambda 650S spectrophotometer (PerkinElmer, Inc., Shelton, CT, USA). The XPS spectra were recorded using a Thermo Fisher Scientific Escalab 250Xi spectrometer (Thermo Scientific™, Waltham, MA, USA).

Work function measurements were performed with a scanning Kelvin probe system SKP5050 (KP Technology, Wick, Scotland) versus a golden reference probe electrode (probe area 2 mm^2^). The probe oscillation frequency was 74 Hz, and the back potential was 7000 mV. Work function values were obtained by averaging 50 data points for four different sites of each sample. Estimated experimental error does not exceed ±0.06 eV.

The contact angle values were measured using optical tensiometer (Bioline Theta Lite, Biolin Scientific, Gothenburg, Sweden). The surface energy was calculated by the Owens–Wendt–Rabel–Kaelble (OWRK)/Fowkes approach using the two-liquid method (water contact angle versus methylene iodide contact angle) [40]. Ultrapure water has initial pH of 5.5. An experimental error of contact angle measurements was determined using 5 data points measured at different spots of the coating and did not exceed 2°.

The work function and contact angle were measured after each step of the surface treatment procedure. After annealing at 200 °C for 30 min, the state of the film surface is designated as “as prepared”. After wetting in the ultrapure water with pH of 5.5 and drying at 80 °C, the surface state is denoted as either “after wetting” or “initial state”. The third step was irradiation of the coatings: the surface state of the samples irradiated by ultraviolet (UV) or visible light is mentioned as “after UV irradiation” or “after Vis irradiation”, respectively.

The irradiation of the films by UV or visible light was carried out using 150 W Xenon lamp (LOMO) equipped with a water filter and UV band pass (250 nm < λ_pass_ < 400 nm) or Vis cutoff color filter (λ_cut_ = 420 nm). The irradiance was 1.19 and 12.3 mW for UV and Vis irradiation, respectively. For all sample surfaces, the kinetics of the photoinduced water contact angle alteration was presented as a dependence of the water contact angle on the irradiation time.

## 3. Results

### 3.1. Surface Characterization

The analysis of experimental data obtained by XRD, XPS, SEM and AFM methods (see the Supplementary Materials) confirmed the formation of coatings with desired structures. Indeed, XRD patterns of metal oxide coatings indicate the presence of the corresponding crystal phases of TiO_2_ (anatase), ZnO (zincite) and Cu_2_O (cuprite) (see Figures S1 and S2).

SEM images demonstrate that coatings were formed by closely packed nanoparticles with average sizes about 50 nm for TiO_2_ and about 15 nm for ZnO (see Figures S3 and S4). AFM data show that the roughness of the coating surfaces does not exceed ±5 nm (see Figures S5 and S6), which indicates a nice smoothness of the surface, and, therefore, the surface hydrophilicity of the coatings is not significantly affected by the surface profile. All major characteristics of the prepared nanocoating surfaces are summarized in Table 1.

### 3.2. Electronic Properties of the Nanocoating Components

**Figure 1:**
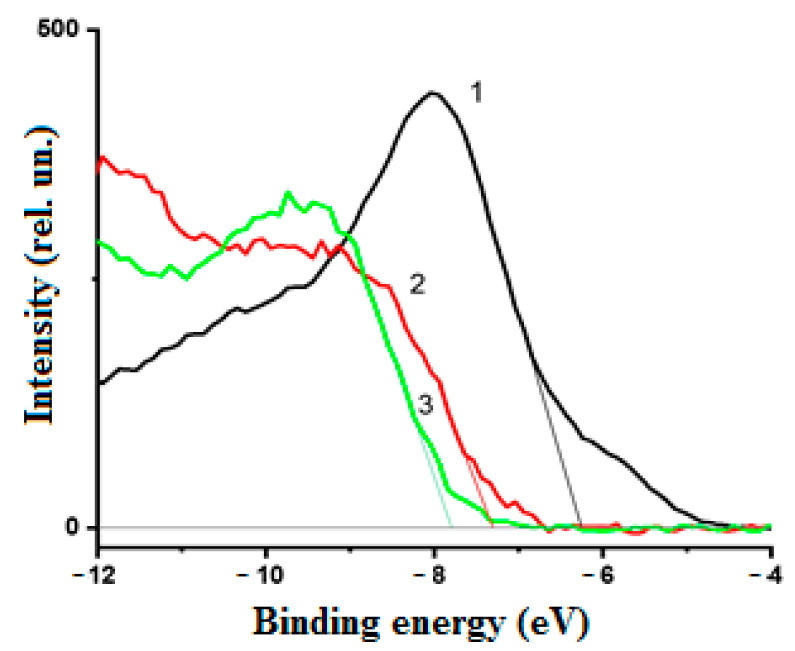
Low binding energy XPS spectra of the components of heterostructured coatings: 1, Cu_2_O; 2, TiO_2_; and 3, ZnO.

XPS spectra recorded in low binding energy region (see Figure 1) were used to determine positions of the valence bands of the components of heterostructured coatings. Corresponding values in vacuum scale are given in Table 2.

The Kelvin probe method was applied to measure work function characteristics of the nanocoatings to estimate the positions of the corresponding Fermi levels. The results of the measurements are given in Table 2. Based on the obtained parameters, one can sketch the energetic diagrams of the components forming heterostructured nanocoating, particularly, assuming that energy level of the bottom of conduction band (E_CB_) is a sum of the energies corresponding to the top of the valence band (E_VB_) and optical band gap energy (E_bg_) of the corresponding components:

E_CB_ = E_VB_ + E_bg_ (7)

**Figure 2:**
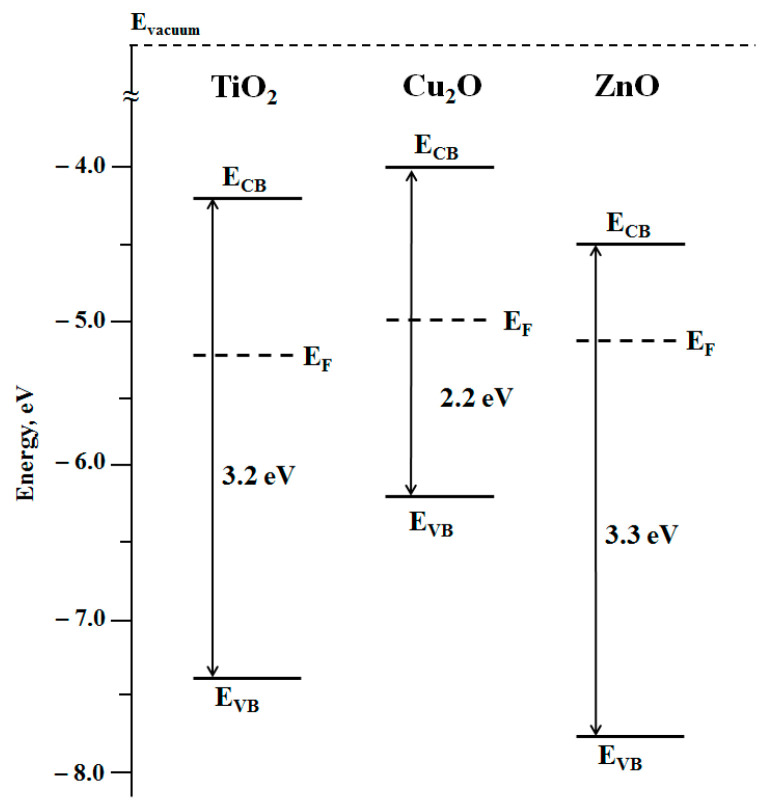
Energy diagrams of the positions of the top of the valence bands and the bottom of the conduction bands and Fermi level positions of the individual components of the heterostructured coatings.

The corresponding energy diagrams of the semiconductor components forming the heterostructured nanocoatings including Fermi level positions are presented in Figure 2.

According to the energy diagrams shown in Figure 2, the formation of Cu_2_O/TiO_2_ and Cu_2_O/ZnO heterostructures should result in electron transfer from Cu_2_O to either TiO_2_ or ZnO, both in the dark and under photoexcitation.

### 3.3. Effect of Light Irradiation on the Photoinduced Hydrophilicity of Heterostructured Nanocoating Surfaces

Electron transfer from Cu_2_O to either TiO_2_ or ZnO in the dark caused by formation of the corresponding heterostructures should lead to the establishment of new equilibrium states of the heterostructures characterizing by new Fermi level positions located between the Fermi level positions of the individual components. Experimental data of new Fermi level positions of Cu_2_O/TiO_2_ and Cu_2_O/ZnO heterostructures indicate a successful formation of heterostructures (see Figure 2 and the experimental data in Table 3). The effect of photoinduced alteration of the surface hydrophilicity of nanocoatings was studied for strongly hydrated surface. As noted in the Experimental Section, such state of the surface is characterized by good reproducibility and considered as the “initial” surface state for studies of photostimulated alteration of the surface hydrophilicity.

Particularly, strong surface wetting leads to alteration of the work function values due to the formation of the multi-layered hydroxyl-hydrated structure of adsorbed water on the surfaces. As evident from experimental data, the effect of surface wetting decreases as follows: TiO_2_ > TiO_2_/Cu_2_O > ZnO > ZnO/Cu_2_O > Cu_2_O. That indicates that the most significant interaction between adsorbed water and metal oxide surface resulting in formation of the surface dipole moment affecting work function takes place on the TiO_2_ surface, while the Cu_2_O surface is practically unaffected by wetting.

**Figure 3:**
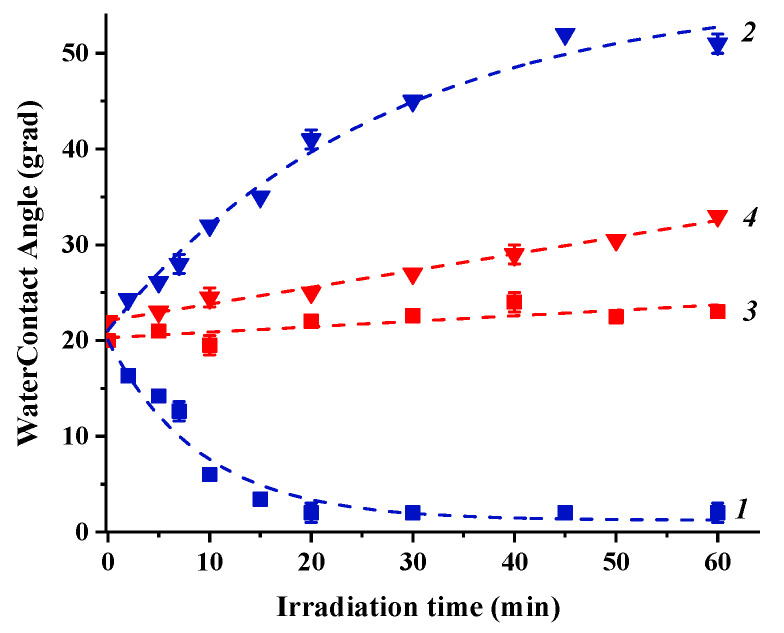
Kinetics of alteration of the water contact angle on the surfaces of TiO_2_ (1 and 3) and TiO_2_/Cu_2_O (2 and 4) coatings under UV (1 and 2) and visible (3 and 4) light irradiation.

**Figure 4:**
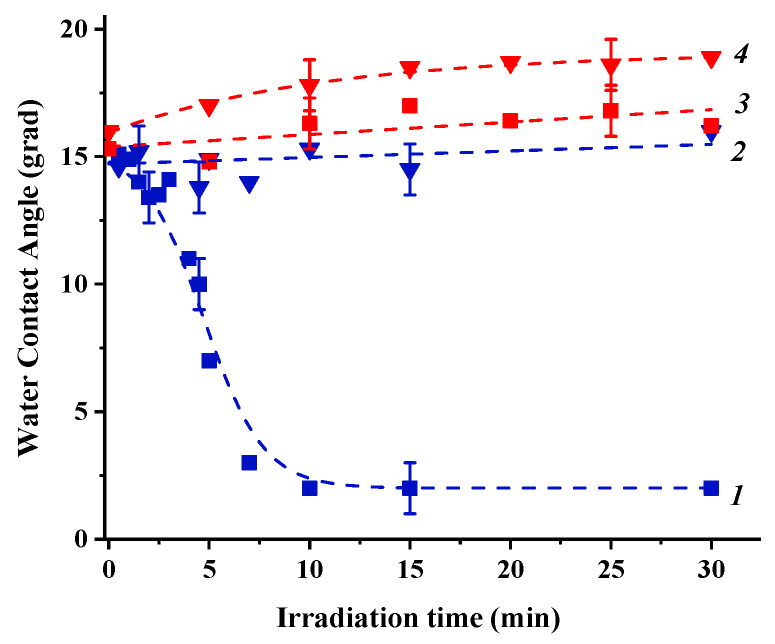
Kinetics of alteration of the water contact angle on the surfaces of ZnO (1 and 3) and ZnO/Cu_2_O (2 and 4) coatings under UV (1 and 2) and visible (3 and 4) light irradiation.

Irradiation of the “initial” state of the coating surfaces with either UV or visible light results in alteration of the surface hydrophilicity (see Figure 3 and Figure 4). To observe the effect of Cu_2_O substrate on photoinduced alteration of the surface hydrophilicity of TiO_2_ and ZnO, the kinetics of water contact angle alteration measured for heterostructured coating are compared with the corresponding kinetics for pristine TiO_2_ and ZnO coatings.

As evident from the presented dependencies, both TiO_2_ and ZnO single component coatings demonstrate a surface conversion to superhydrophilic states under UV irradiation and no significant alteration of the surface hydrophilicity under visible light irradiation. However, the presence of the Cu_2_O substrate in the heterostructured coatings drastically changes the photoinduced hydrophilic behavior of both TiO_2_ and ZnO surfaces. Indeed, under UV irradiation, both surfaces do not demonstrate any tendency to transform to superhydrophilic state.

Conversely, both surfaces show decrease of the surface hydrophilicity: stronger for TiO_2_ and weaker for ZnO. At the same time, a pronounced decrease of the surface hydrophilicity is also observed under visible light irradiation for both surfaces when neither TiO_2_ nor ZnO is photoexcited but Cu_2_O only (see data on their band gaps in Table 2). In general, a main reason and a driving force for alteration of the surface hydrophilicity is the alteration of the surface energy. Therefore, we estimated the total surface energy and its polar and dispersion components using the “two-liquid” approach. The corresponding values of the total surface free energy and its polar and dispersion components for the “initial” surface states of the heterostructured coatings and ZnO and TiO_2_ coatings and after UV and visible light irradiation are presented in Table 4. The graphical presentation of the tendencies of SFE alteration caused by irradiation is shown in Figures S9–S11.

Thus, as evident from the presented data, the transformation of TiO_2_ and ZnO surfaces to superhydrophilic state caused by UV irradiation is accompanied by an increase of the total surface free energy with a major impact from its polar component for TiO_2_ and from its dispersive component for ZnO, while visible light irradiation induced only minor alteration of the total surface free energy and its components, which results in very weak changes in the surface hydrophilicity. At the same time, the surfaces of both Cu_2_O/TiO_2_ and Cu_2_O/ZnO heterostructured coatings demonstrate a significant decay of the total surface energy caused by both UV and visible light irradiation, resulting in a decrease of the surface hydrophilicity. Note that the impact of the alteration of polar and dispersive components on changes of the total surface energy and surface hydrophilicity for heterostructured coatings is different for ZnO and TiO_2_ surfaces and depends on the spectral region of photoexcitation. Indeed, UV irradiation of the Cu_2_O/TiO_2_ system results in decay of both polar and dispersive components of TiO_2_ total surface energy, while, for Cu_2_O/ZnO, the major impact on the surface energy decay originates from the decrease of the dispersive component, which completely compensates the slight increase of the polar component. At the same time, visible light irradiation (resulting in photoexcitation of Cu_2_O only) causes mainly the decrease of the dispersive component for Cu_2_O/TiO_2_ heterostructure and the decay of the polar component for Cu_2_O/ZnO system.

In turn, the alteration of the work function values induced by either UV or visible light irradiation (see Table 3 and Figure S12) demonstrates strong decrease of the work function for Cu_2_O/TiO_2_ coating and practically no significant alteration for Cu_2_O/ZnO system.

## 4. Discussion

**Figure 5:**
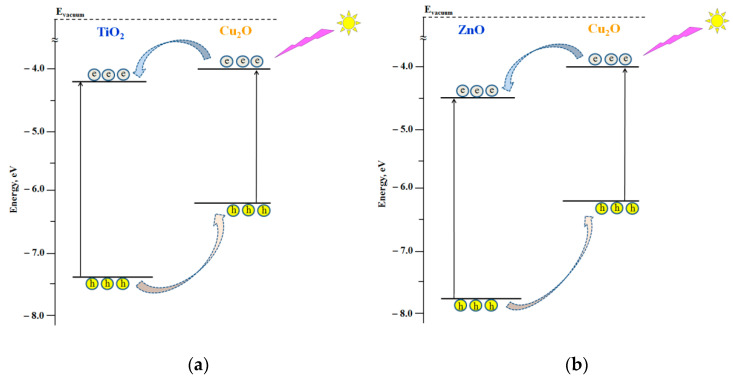
Schemes of the electron transfer in heterostructured coatings under UV light photoexcitation: (**a**) Cu_2_O/TiO_2_ and (**b**) Cu_2_O/ZnO.

**Figure 6:**
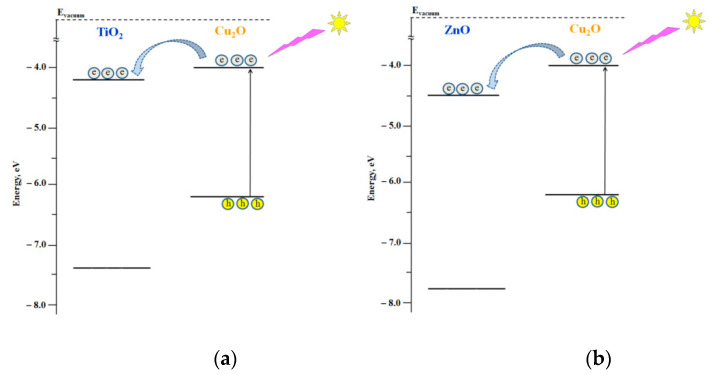
Schemes of the electron transfer in heterostructured coatings under visible light photoexcitation: (**a**) Cu_2_O/TiO_2_ and (**b**) Cu_2_O/ZnO.

Both Cu_2_O/TiO_2_ and Cu_2_O/ZnO systems represent type II heterostructures, which provides a condition for charge separation at the corresponding heterojunctions, particularly, electron transfer from Cu_2_O to either TiO_2_ or ZnO (see Figure 5 and Figure 6).

Consequently, both UV and visible light irradiation of heterostructures results in the increase of the electron concentration compared to the concentration of holes in both TiO_2_ and ZnO. According to our proposed model described in the Introduction (see Equations (1)–(6)), the alteration of the ratio between electrons and holes at the surface, *n*_1_/*n*_2_, can result in a significant alteration of the surface hydrophilicity and turn it, particularly, from a more hydrophilic state to a less hydrophilic state. Therefore, we may conclude that a higher concentration of electrons on the TiO_2_ and ZnO surfaces induced by electron transfer from Cu_2_O results in the surface transformation to a less hydrophilic state.

Remarkably, the formation of Cu_2_O/TiO_2_ and Cu_2_O/ZnO heterostructures also strongly affects the surface free energy behavior under their photoexcitation. Indeed, UV irradiation of the individual TiO_2_ and ZnO surfaces leads to an increase of the SFE and, particularly, its polar component, which is a main reason for the surface transformation to the superhydrophilic state. However, photoexcitation of heterostructures and electron transfer from Cu_2_O to either TiO_2_ or ZnO results in the decay of the total SFE and its polar component. This observation may indicate that excess of electrons at the coating surfaces can destroy the order of the hydroxyl-hydrated multi-layer structure of adsorbed water and, thus, decrease the total surface energy of the system, resulting in a decreasing of the surface hydrophilicity. This conclusion was confirmed by the effect of visible light irradiation when only the Cu_2_O component of the heterostructures was excited, and, therefore, only electron transfer from Cu_2_O to TiO_2_ and ZnO components could affect both SFE and surface hydrophilicity. Photoinduced destruction of the order in the hydroxyl-hydrated multi-layer structure of adsorbed water is also (indirectly) confirmed by the decrease of the work function of the heterostructures under irradiation, which might be caused by a decrease of the surface dipole moment due to the disorder of the adsorbed water structure in the hydroxyl-hydrated multilayer.

Summing up, we may conclude that the electron transfer realized in Cu_2_O/TiO_2_ and Cu_2_O/ZnO heterostructures under photoexcitation leads to an increase of the concentration of electrons on the TiO_2_ and ZnO surfaces that causes a higher disorder in the hydroxyl-hydrated multi-layer structure of the adsorbed water, thus resulting in the decay of the SFE and the surface hydrophilicity.

## 5. Conclusions

We demonstrated the effect of Cu_2_O substrate on the effect of the photoinduced alteration of the surface hydrophilicity of TiO_2_ and ZnO surfaces under both UV and visible light irradiation. Particularly, it was shown that under UV light both surfaces can be transformed to the superhydrophilic state, while both UV and visible light irradiation of the heterostructured coating results in a decrease of the surface hydrophilicity. This effect of the Cu_2_O substrate on the photoinduced hydrophilic behavior of TiO_2_ and ZnO surfaces is explained in terms of charge separation at corresponding heterojunctions and enrichments of TiO_2_ and ZnO surfaces with electrons transferred from Cu_2_O. Consequently, we may conclude that an increase of the free electron concentration on the surface of both TiO_2_ and ZnO leads to the decay of their surface hydrophilicity. Thus, the purposeful formation of a certain type heterostructures may help to control the surface hydrophilicity at the desired level and the direction of its photostimulated alteration.

## Figures and Tables

**Table 1:** Characterization of the morphology.

Sample	Crystalline Phase	Thickness, nm	Particle Diameter, nm	Smoothness, nm
TiO_2_	Anatase	55	50	3
TiO_2_/Cu_2_O	Anatase/Cuprite	45/85	40	3
ZnO	Zincite	100	15	4
ZnO/Cu_2_O	Zincite /Cuprite	120/80	15	5

**Table 2:** Positions of the conduction and valence bands and band gap energies of the coating components with respect to vacuum energy level.

Sample	E_bg_ *, eV	E_VB_, eV	E_CB_, eV	E_F_, eV
TiO_2_	3.2	−7.4	−4.2	−5.24
ZnO	3.3	−7.8	−4.5	−5.14
Cu_2_O	2.2	−6.2	−4.0	−5.04

* The E_bg_ values were determined using transmittance spectra of nanocoatings and corresponding Tauc plots (see Figures S7 and S8).

**Table 3:** Work function values (WF) after different treatments for all studied coatings.

Coatings	WF, eV as Prepared	WF, eV after Wetting	WF, eV after UV-Irradiation	WF, eV after Vis Irradiation
Cu_2_O	5.04	4.94	4.99	5.14
TiO_2_	5.24	6.79	5.40	6.92
TiO_2_/Cu_2_O	5.17	5.64	4.59	4.98
ZnO	5.14	5.49	4.99	5.64
ZnO/Cu_2_O	5.09	5.35	5.06	5.36

**Table 4:** Total (t), polar (p), and dispersive (d) surface free energies (SFE) for all coatings studied after different treatments.

SFE, mJ/m^2^	After Wetting			After UV Irradiation			After Vis Irradiation		
	t	p	d	t	p	d	t	p	d
TiO_2_	73.4	42.9	31.4	79.7	47.3	32.4	74.1	42.5	31.6
TiO_2_/Cu_2_O	74.4	41.6	32.8	56.3	30.3	26	67.8	42.9	24.9
ZnO	74.0	46.2	27.8	78.3	44.2	34.1	71.5	45.4	26.1
ZnO/Cu_2_O	75.3	44.2	31.1	73.7	47.2	26.5	71.4	39.6	31.8

## Supplementary Materials

The following are available online at https://www.mdpi.com/article/10.3390/nano11061526/s1, Figure S1: XRD patterns of Cu_2_O, TiO_2_ and TiO_2_/Cu_2_O heterostructured coatings, Figure S2: XRD patterns of Cu_2_O, ZnO and ZnO/Cu_2_O heterostructured coatings, Figure S3: SEM images of the TiO_2_ surface and TiO_2_/Cu_2_O cross-section of the TiO_2_/Cu_2_O coating, Figure S4: SEM images of the TiO_2_ surface and TiO_2_/Cu_2_O cross-section of the TiO_2_/Cu_2_O coating, Figure S5: AFM image of the TiO_2_ surface and roughness profile of the TiO_2_/Cu_2_O coating, Figure S6: AFM image of the ZnO surface and roughness profile of the ZnO/Cu_2_O coating, Figure S7: Transmittance spectra of: (a) TiO_2_/Cu_2_O heterostructured coating and its components; and (b) ZnO/Cu_2_O heterostructured coating and its components, Figure S8: Tauc plots for 1 – Cu_2_O, 2 – ZnO, and 3 – TiO_2_., Figure S9: Alteration of the total SFE caused by irradiation with visible and UV light, Figure S10: Alteration of the SFE polar component caused by irradiation with visible and UV light, Figure S11: Alteration of the SFE dispersive component caused by irradiation with visible and UV light, Figure S12: Alteration of the work function of nanocoatings induced by wetting and UV and visible light irradiation.
