# Supplementary material for: Effect of Cu2O Substrate on Photoinduced Hydrophilicity of TiO2 and ZnO Nanocoatings
Source: Nanomaterials (Basel). 2021 Jun 9;11(6):1526. doi: 10.3390/nano11061526 (PMC8226581; doi:10.3390/nano11061526)
Supplement: Supplementary file 1 [file nanomaterials-11-01526-s001.zip › nanomaterials-1230881-SI-final-6.24.pdf]

nanomaterials

Supporting information

Effect of Cu_2O Substrate on Photoinduced Hydrophilicity of TiO_2 and ZnO Nanocoatings

Maria V. Maevskaya [1], Aida V. Rudakova [1], Alexei V. Emeline [1] and Detlef W. Bahnemann [1,2,*]

[1] Laboratory "Photoactive Nanocomposite Materials", Saint-Petersburg State University, Ulianovskaia str. 1, Peterhof, 198504 Saint-Petersburg, Russia; maevskaya.mv@gmail.com (M.V.M.); Aida.Rudakova@spbu.ru (A.V.R.); alexei.emeline@spbu.ru (A.V.E.)

[2] Institut fuer Technische Chemie, Gottfried Wilhelm Leibniz Universitaet Hannover, Callinstrasse 3, D-30167 Hannover, Germany

* Correspondence: bahnemann@iftc.uni-hannover.de

XRD data

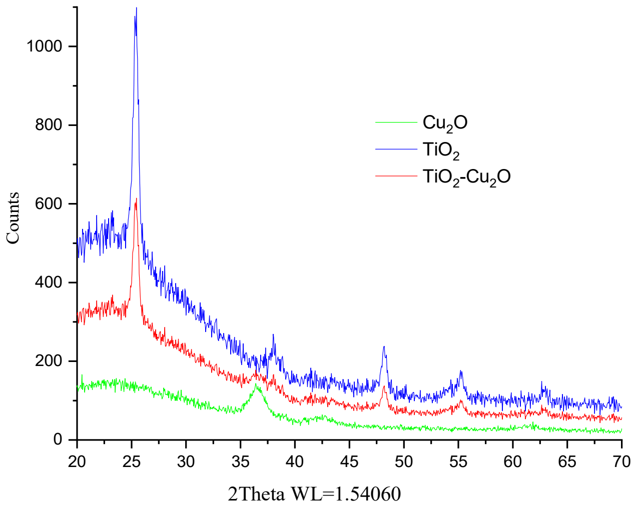

Figure S1. XRD patterns of Cu_2O, TiO_2 and TiO_2/Cu_2O heterostructured coatings.

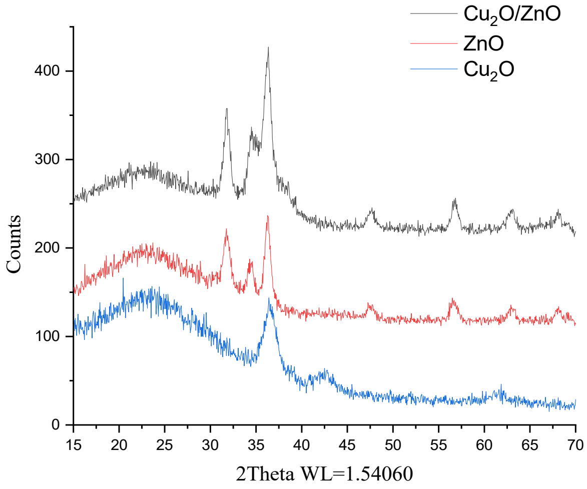

Figure S2. XRD patterns of Cu_2O, ZnO and ZnO/Cu_2O heterostructured coatings.

SEM images of the sample surfaces and cross-sections

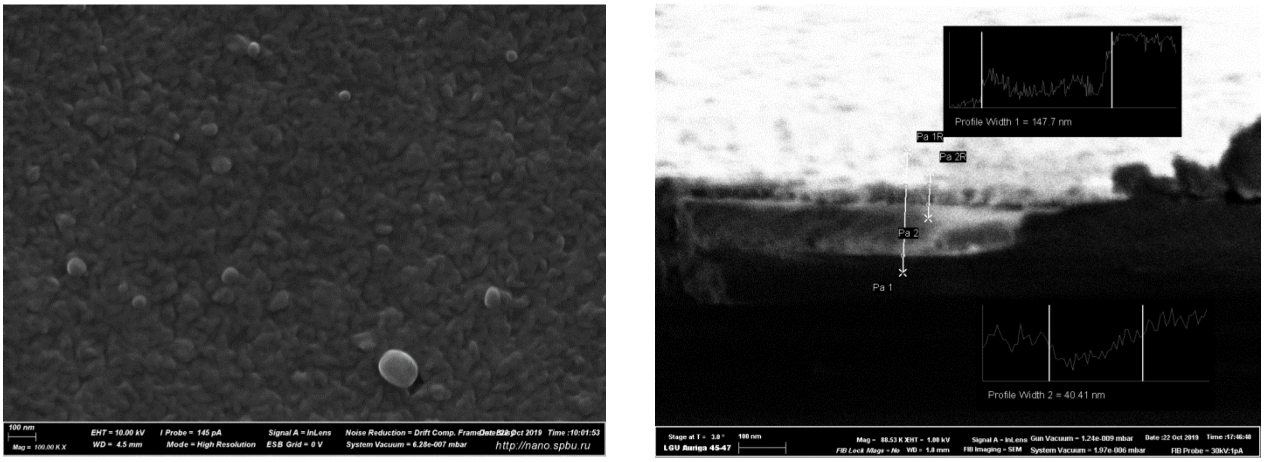

Figure S3. SEM images of the TiO_2 surface and TiO_2/Cu_2O cross-section of TiO_2/Cu_2O coating.

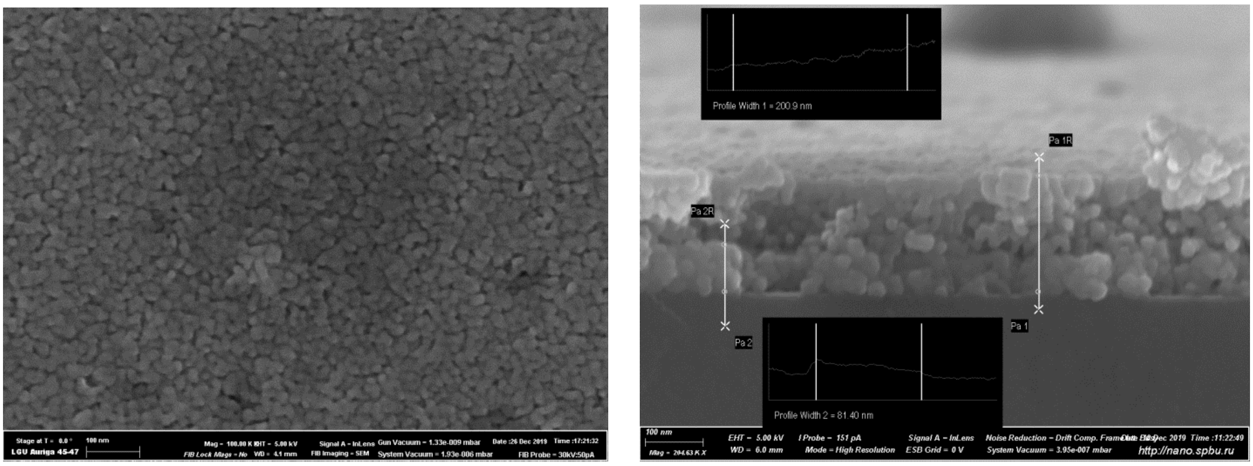

Figure S4. SEM images of the TiO_2 surface and ZnO/Cu_2O cross-section of ZnO/Cu_2O coating.

AFM image of the surfaces and roughness profiles

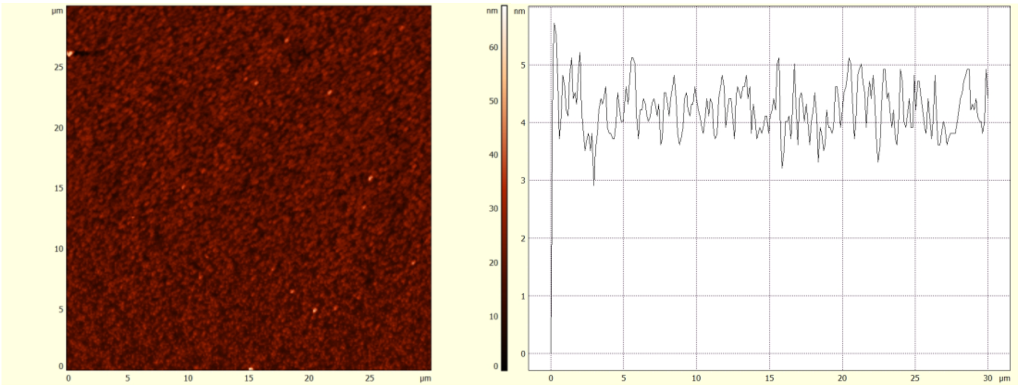

Figure S5. AFM image of the TiO_2 surface and roughness profile of TiO_2/Cu_2O coating.

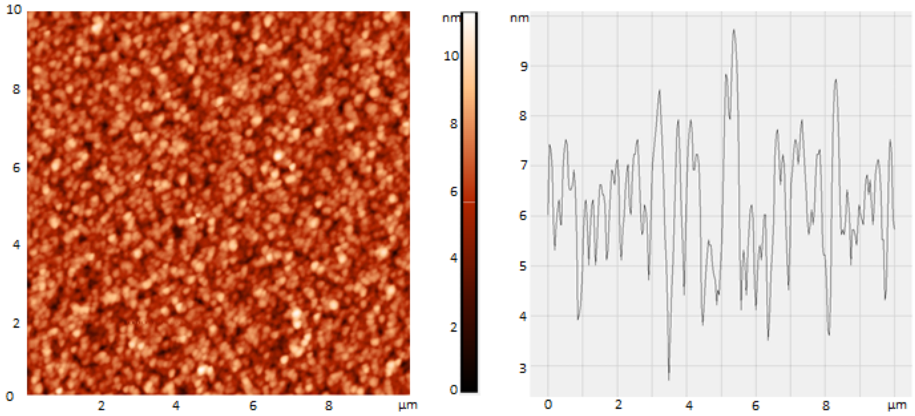

Figure S6. AFM image of the ZnO surface and roughness profile of ZnO/Cu_2O coating.

Transmittance spectra of individual components and heterostructures.

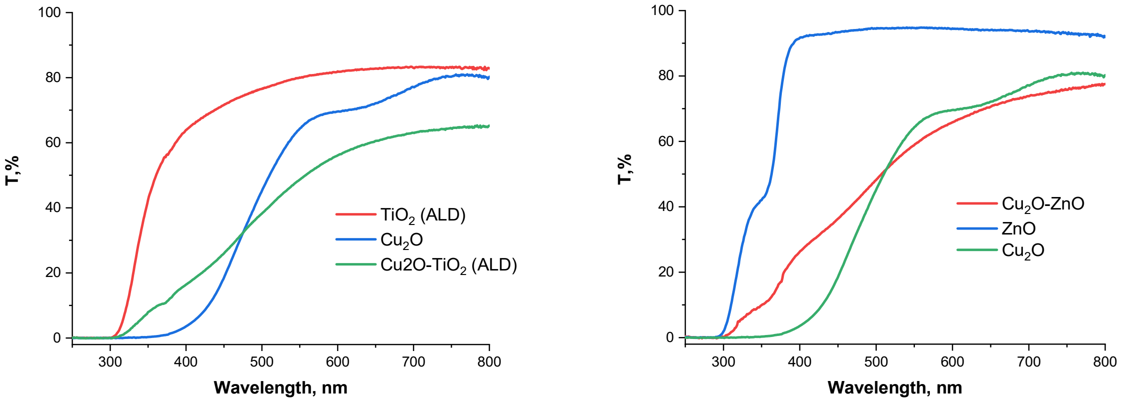

(**a**) (**b**)

Figure S7. Transmittance spectra of (**a**) TiO_2/Cu_2O heterostructured coating and its components, and (**b**) ZnO/Cu_2O heterostructured coating and its components.

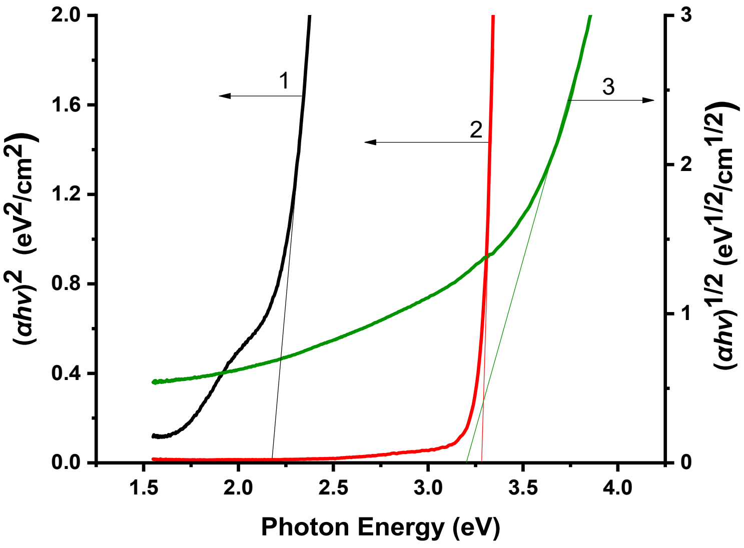

Figure S8. Tauc plots for 1 – Cu_2O, 2 – ZnO, and 3 – TiO_2.

Alteration of SFE and its polar and dispersive components.

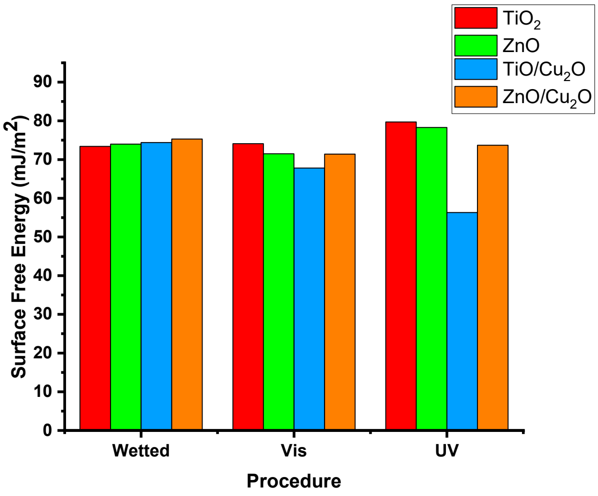

Figure S9. Alteration of the total SFE caused by irradiation with visible and UV light.

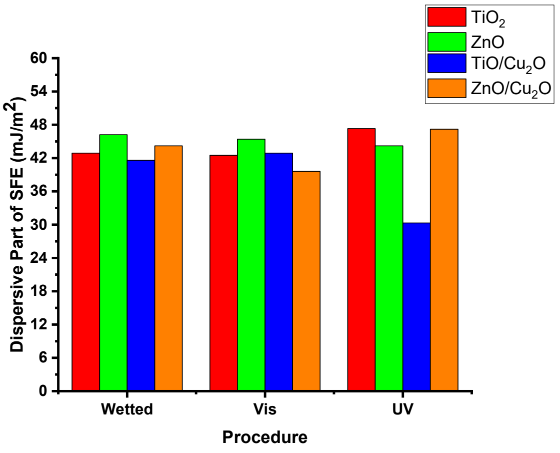

Figure S10. Alteration of the SFE polar component caused by irradiation with visible and UV light.

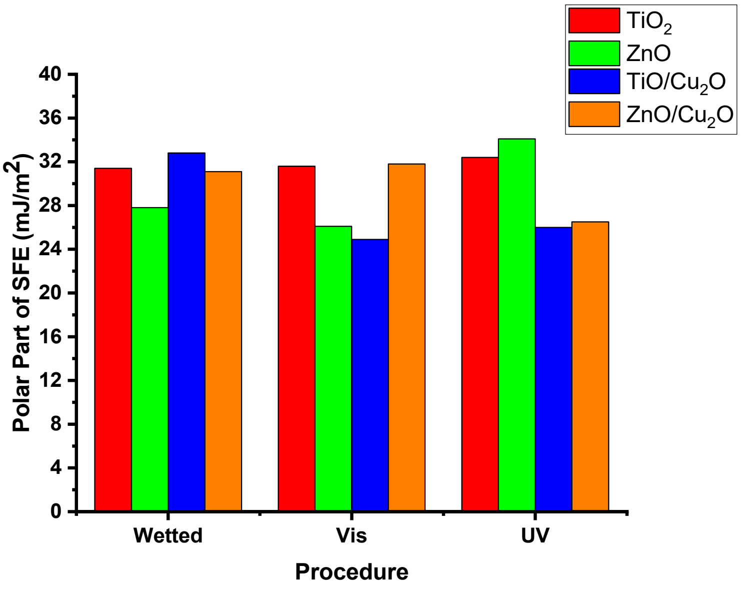

Figure S11. Alteration of the SFE dispersive component caused by irradiation with visible and UV light.

Alteration of the work function of nanocoatings induced by wetting and UV and visible light irradiation.

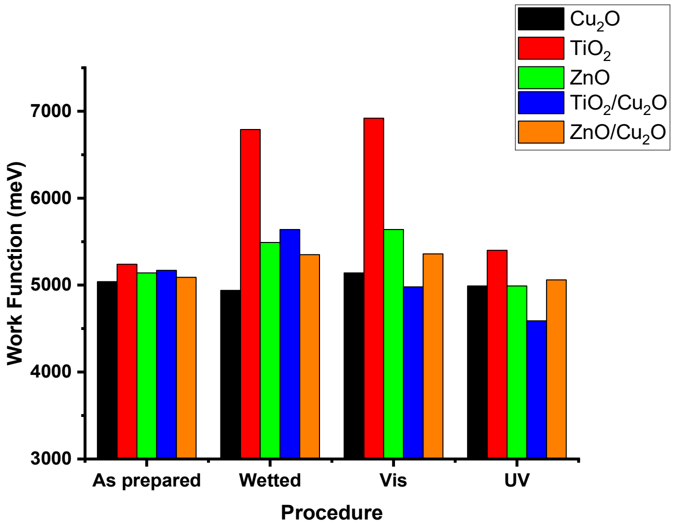

Figure S12. Alteration of the work function of nanocoatings induced by wetting and UV and visible light irradiation.
